# Mediterranean Journal of Rheumatology March 2019 Highlights

**DOI:** 10.31138/mjr.30.1.1

**Published:** 2019-03-28

**Authors:** George D. Kitas

**Affiliations:** Departments of Rheumatology and Research and Development, Dudley Group NHS Foundation Trust (Teaching Trust of the University of Birmingham, UK), Russells Hall Hospital, Dudley, West Midlands, UK; Arthritis Research UK Epidemiology Unit, University of Manchester, Manchester, UK

Welcome to the first issue of MJR for 2019: it hosts interesting contributions from authors across the Mediterranean area and beyond. In this issue we bring together facets of rheumatology clinical care and aspects of laboratory techniques used in the evaluation and validation of immunological tests.

In their editorial, Bragazzi et al.^1^ discuss how data originating from large population-based databases could contribute to the decision making and improve clinical practice. They comment on the study of Mansour et al.,^2^ who collected data from a large health provider organization in Israel. The authors look back to the first steps of rheumatology and present how data collection in this field has evolved over the last 150 years.

A top-quality review article by Nikolopoulos et al.^3^ discusses the serious problem of cerebrovascular events in patients with Systemic Lupus Erythematosus (SLE). Stroke is the major central nervous system manifestation of SLE: Boumpas and his team, world experts in the field of SLE, provide comprehensive information about the epidemiology, work-up, management and primary prevention of cerebrovascular events in patients with lupus. The role of thrombolysis as a therapeutic intervention in this particular group of patients is also highlighted in the article.

Systemic sclerosis (SSc)-related interstitial lung disease remains an extremely challenging area in rheumatology with significant advances and numerous ongoing trials investigating regimens with different modes of action, namely conventional and biologic immunosuppressants, anti-fibrotic agents, and cannabinoid receptors. Daoussis & Liossis, who have produced original data of their own in this field, summarise the recent data and provide practical guidance regarding the treatment of this difficult-to-manage subgroup of SSc patients, focusing on mycophenolate mofetil as the cornerstone of treatment for patients with severe pulmonary involvement.^4^

Monogioudi and Zegers from the European Commission Joint Research Centre in Belgium remind us how important, but also how hard, is the process of validation and standardization of autoantibody testing techniques to ensure reliable results and correct diagnosis in autoimmune diseases across different countries and areas. The authors underline the collaboration between the European Commission and scientific organizations in the development of autoantibody assays.^6^

Besides systemic diseases, in daily clinical rheumatology practice we are frequently confronted with syndromes with diffuse or residual musculoskeletal pain requiring a multidisciplinary approach by different medical teams and allied health professionals. Complex regional pain syndrome is a typical difficult-to-treat disease: in this issue Misidou & Papagoras provide an excellent clinical update of this condition with practical tips regarding its overall management.^5^ They underline the importance of physical therapy and rehabilitation, which are at least as important as the various pharmacological interventions.

The risk of venous thromboembolic events in patients with autoimmune disorders has attracted the interest of rheumatologists over the last years. In the current issue Mansour et al. present a population-based study from the largest health provider in Israel assessing the incidence of deep venous thrombosis and pulmonary embolism in 11,782 rheumatoid arthritis (RA) patients and 57,973 age- and gender-matched controls.^2^ In line with previous reports, they demonstrated an increased risk of deep vein thrombosis in RA patients which was associated with high CRP levels. These findings confirm once more the tight link between inflammation and vascular disease and underscore the continuous need for optimal control of RA.

A complex case of an elderly female patient with microscopic polyangiitis and pulmonary fibrosis is presented by Koutsoviti et al.^7^ Interstitial lung fibrosis has been recognized recently as a possible initial manifestation of p-ANCA-associated vasculitis, and such cases could promote awareness amongst rheumatologists and other physicians of this type of pulmonary involvement in this specific subgroup of patients with systemic necrotizing vasculitis.

In our regular research protocol section, we publish in this issue 2 protocols funded by the Greek Rheumatology Society and Professional Association of Rheumatologists after external international peer review. They include a prospective study assessing nailfold capillaroscopy as a prognostic tool for the long-term outcome in SSc patients^8^ and the performance of the Assessment of Spondyloarthritis International Society criteria for the classification of ankylosing spondylitis in a large cohort from a regional centre in Crete.^9^

## References

[1] Bragazzi NL Damiani G Martini M From Rheumatology 1.0 to Rheumatology 4.0 and beyond: the contributions of Big Data to the field of rheumatology. Mediterr J Rheumatol 2019;30(1):3–6. 31938766 10.31138/mjr.30.1.3 PMC6959971

[2] Mansour R Azrielant S Watad A Tiosano S Yavne Y Comaneshter D Cohen AD Amital H Venous thromboembolism events among RA patients. Mediterr J Rheumatol 2019;30(1):38–43. 10.31138/mjr.30.1.38 PMC7045915 32185341

[3] Nikolopoulos D Fanouriakis A Boumpas DT Cerebrovascular Events in Systemic Lupus Erythematosus: Diagnosis and Management. Mediterr J Rheumatol 2019;30(1):7–15. 10.31138/mjr.30.1.7 PMC7045913 32185337

[4] Daoussis D Liossis SN Treatment of systemic sclerosis associated fibrotic manifestations: Current options and future directions. Mediterr J Rheumatol 2019;30(1):33–7. 10.31138/mjr.30.1.33 PMC7045920 32185340

[5] Misidou C Papagoras C Complex Regional Pain Syndrome: An update. Mediterr J Rheumatol 2019;30(1):16–25. 10.31138/mjr.30.1.16 PMC7045919 32185338

[6] Monogioudi E Zegers I Certified Reference Materials and their need for the diagnosis of autoimmune diseases. Mediterr J Rheumatol 2019;30(1):26–32. 10.31138/mjr.30.1.26 PMC7045914 32185339

[7] Koutsoviti S Elezoglou A Katsimpari C Sofianos I Raftakis I Theotikos E Samaras C Myriokefalitakis I Pulmonary fibrosis and microscopic polyangiitis in a 75-year-old woman. Mediterr J Rheumatol 2019;30(1):44–7. 10.31138/mjr.30.1.44 PMC7045917 32185342

[8] Repa A Avgoustidis N Kougkas N Bertsias G Zafiriou M Sidiropoulos P Nailfold. Videocapillaroscopy as a Candidate Biomarker for Organ Involvement and Prognosis in Patients with Systemic Sclerosis. Mediterr J Rheumatol 2019;30(1):48–50. 10.31138/mjr.30.1.48 PMC7045911 32185343

[9] Kougkas N Avgoustidis N Repa A Bertsias G Eskitzis A Sidiropoulos P The value of the 2011 ASAS classification criteria in patients with Spondyloarthritis and the prognosis of non-radiographic axial Spondyloarthritis: data from a large cohort of a tertiary referral hospital. Mediterr J Rheumatol 2019;30(1):51–3. 10.31138/mjr.30.1.51 PMC7045912 32185344

